# Audit of Data Sharing by Pharmaceutical Companies for Anticancer Medicines Approved by the US Food and Drug Administration

**DOI:** 10.1001/jamaoncol.2022.2867

**Published:** 2022-07-28

**Authors:** Natansh D. Modi, Ahmad Y. Abuhelwa, Ross A. McKinnon, Alan V. Boddy, Mark Haseloff, Michael D. Wiese, Tammy C. Hoffmann, Eric D. Perakslis, Andrew Rowland, Michael J. Sorich, Ashley M. Hopkins

**Affiliations:** 1 College of Medicine and Public Health, Flinders University, Adelaide, South Australia, Australia; 2 Clinical and Health Sciences, University of South Australia, Adelaide, South Australia, Australia; 3 Faculty of Health Sciences and Medicine, Bond University, Gold Coast, Queensland, Australia; 4 Duke Forge, Duke University Medical Center, Durham, North Carolina

## Abstract

**Importance:**

Emerging policies drafted by the pharmaceutical industry indicate that they will transparently share clinical trial data. These data offer an unparalleled opportunity to advance evidence-based medicine and support decision-making.

**Objective:**

To evaluate the eligibility of independent, qualified researchers to access individual participant data (IPD) from oncology trials that supported US Food and Drug Administration (FDA) approval of new anticancer medicines within the past 10 years.

**Design, Setting, and Participants:**

In this quality improvement study, a cross-sectional analysis was performed of pivotal clinical trials whose results supported FDA-approved anticancer medicines between January 1, 2011, and June 30, 2021. These trials’ results were identified from product labels.

**Exposures:**

Eligibility for IPD sharing was confirmed by identification of a public listing of the trial as eligible for sharing or by receipt of a positive response from the sponsor to a standardized inquiry.

**Main Outcomes and Measures:**

The main outcome was frequency of IPD sharing eligibility. Reasons for data sharing ineligibility were requested and collated, and company-, drug-, and trial-level subgroups were evaluated and presented using χ^2^ tests and forest plots.

**Results:**

During the 10-year period examined, 115 anticancer medicines were approved by the FDA on the basis of evidence from 304 pharmaceutical industry–sponsored trials. Of these trials, 136 (45%) were eligible for IPD sharing and 168 (55%) were not. Data sharing rates differed substantially among industry sponsors, with the most common reason for not sharing trial IPD being that the collection of long-term follow-up data was still ongoing (89 of 168 trials [53%]). Of the top 10 anticancer medicines by global sales, nivolumab, pembrolizumab, and pomalidomide had the lowest eligibility rates for data sharing (<10% of trials).

**Conclusions and Relevance:**

There has been a substantial increase in IPD sharing for industry-sponsored oncology trials over the past 5 years. However, this quality improvement study found that more than 50% of queried trials for FDA-approved anticancer medicines were ineligible for IPD sharing. Data accessibility would be substantially improved if, at the time of FDA registration of a medicine, all data that support the registration were made available.

## Key Points

**Question:**

What proportion of clinical trials that underpin registration of contemporary anticancer medicines are eligible for individual participant data (IPD) sharing with qualified researchers?

**Findings:**

In this quality improvement study of the 304 trials that underpinned the US Food and Drug Administration (FDA) registration of 115 anticancer medicines over the past 10 years, 136 (45%) were eligible for IPD sharing.

**Meaning:**

Although inroads have been made toward improving IPD transparency over the past 5 years, these findings suggest that a substantial portion of pivotal oncology trials that support the FDA registration of modern anticancer medicines remain unavailable to qualified researchers.

## Teaser

This quality improvement study audits individual participant data sharing by pharmaceutical companies for anticancer medicines approved by the US Food and Drug Administration.

## Introduction

Decisions by regulators and clinicians on whether to approve and use new medications are typically based on findings from pivotal clinical trials.^1^ For most newer medicines, an industry sponsor drives the early generation of the evidence base supporting the medicine, but this process requires access to and facilitation by global health care systems.^2^ Data from early clinical trials remain the centerpiece of safety and efficacy assessments, at least until postmarketing data can reach maturity.^3,4^ Transparent sharing of individual participant data (IPD) from clinical trials facilitates enrichment of the postapproval evidence base through novel secondary analyses and informs the design of future studies.^1,4,5,6,7,8,9,10^

In 2010, the European Medicines Agency began adopting forward-looking policies that promote clinical trial data sharing at the time of market authorization.^1^ In 2014, the pharmaceutical industry, via the Pharmaceutical Research and Manufacturers of America (PhRMA) and the European Federation of Pharmaceutical Industries and Associations (EFPIA), acknowledged the importance of IPD sharing and endorsed a commitment to sharing anonymized IPD for approved medicines upon request by qualified researchers.^11,12^ A 2018 audit reported that data from only 15% of clinical trials were available for sharing 2 years after publication of the primary outcome, with no sharing occurring for oncology trials.^13^ Since 2018, there has been substantial development of resources and systems to facilitate research using transparently shared IPD,^5,14,15,16,17,18,19^ and progress has been made by the pharmaceutical industry to develop data sharing policies and processes. Thus, the status of IPD sharing of pivotal oncology trials warrants reevaluation.

Sharing of IPD is critical for highly used newer medicines when the evidence underpinning their use is almost exclusively derived from clinical trials supporting the medicines’ approval. Despite being one of the most active areas for drug development over the past decade, there is limited data regarding the sharing of anonymized IPD underlying pivotal oncology trials for newer anticancer medicines. This study evaluated the eligibility of independent, qualified researchers to access IPD from oncology trials that supported the US Food and Drug Administration (FDA) approval of new anticancer medicines within the past 10 years.

## Methods

### Sample and Data

For this quality improvement study, a structured search was undertaken to identify all anticancer medicines approved by the FDA between January 1, 2011, and June 30, 2021.^20,21^ For these anticancer medicines, product labels were accessed through the National Institutes of Health DailyMed database,^22^ and a list of the clinical trials that had their results summarized in the product labels was made. This research is of negligible risk and therefore exempt from institutional review board review. This study followed the Standards for Quality Improvement Reporting Excellence (SQUIRE) reporting guideline.

For each trial, information on the National Clinical Trial number, phase of the trial, the date trial results were added to the product label, and owner of the medicine was collected. Information on the primary sponsor, cancer type (solid or hematologic), start date, primary completion date, and final completion date was collected from ClinicalTrials.gov. For industry-sponsored trials, data were collated on whether the sponsoring pharmaceutical company (ie, the owner of the medicine) was within the top 20 by global revenue^23,24^ and whether the investigated medicines were within the top 10 anticancer medicines by global sales.^25,26^ The PhRMA and/or EFPIA membership status of trial sponsors was documented, and the websites of trial sponsors were searched to identify the presence of a public IPD sharing policy. The data sharing policies were used to collate information on the data sharing process (ie, a company internal or external process [eg, Vivli,^14^ Clinical Study Data Request,^15^ or Yale University Open Data Access^16^]), contact details for IPD sharing inquiries, and whether trial completion was a criterion for data sharing (ie, whether the policy contains a statement that IPD would not be available until after cessation of follow-up data collection).

### Determination of IPD Sharing Eligibility

Beginning August 1, 2021, the IPD sharing eligibility of each trial was confirmed by either identification of a public listing of the trial as eligible for IPD sharing or receipt of a positive response to a standardized inquiry (eAppendix 1 in Supplement 1) directed to the trial sponsor (or medicine owner if different). Ineligibility for IPD sharing was confirmed by a negative response to the inquiry (ie, receipt of written confirmation from the trial sponsor that IPD would not be shared with independent researchers). If a trial was indicated as not eligible for IPD sharing, details of the reasons for ineligibility and when the trial would become eligible were requested. If no response to the initial inquiry was received, prompts were sent 30 and 60 days later. If no response had been received from the trial sponsor (or medicine owner) by 120 days from the initial inquiry, then the trial was deemed to be ineligible for IPD sharing.

### Statistical Analysis

All statistical analyses were performed using R, version 4.1.2 software (R Foundation for Statistical Computing). Differences in trial IPD sharing eligibility proportions according to key descriptive company-, drug-, and trial-level subgroups were evaluated and presented using χ^2^ tests and forest plots. Statistical significance was set at 2-sided *P* < .05.

## Results

### Sample

During the 10-year sampling period, 115 anticancer medicines were approved by the FDA, of which 96 (83%) are also currently approved by the European Medicines Agency. These medicines were owned or co-owned by 49 pharmaceutical companies, and their approval was based on the results of 304 industry-sponsored trials. All trials were registered through ClinicalTrials.gov. Of the 304 trials, 16 (5%) evaluated cytotoxic medicines; 12 (4%), hormonal medicines; 80 (26%), immunomodulators; and 196 (64%), targeted therapeutics not elsewhere specified. eTable 1 in Supplement 1 provides a detailed summary of each trial by cancer subtype. Of the 304 trials, 203 (67%) included patients with solid tumors, and 101 (33%) included patients with hematologic malignant neoplasms. There were 199 (65%) randomized trials and 105 (35%) nonrandomized trials, including 16 (5%) phase 1, 112 (37%) phase 2, and 176 (58%) phase 3 trials. Of the 304 trials, 140 (46%) had a trial start date before January 1, 2014, and 164 (54%) had a trial start date after January 1, 2014. Less than 3 years had passed since the results summary was added to the product label for 136 (45%) trials, 3 to 7 years for 126 (41%) trials, and more than 7 years for 42 (14%) trials.

Of the 49 pharmaceutical companies audited, 24 were PhRMA/EFPIA members, and 28 had a publicly available IPD sharing policy; these companies sponsored 261 (86%) and 273 (90%) of the trials audited, respectively. Nineteen pharmaceutical companies that sponsored 211 trials (69%) shared IPD through an external platform (Vivli, 13 companies; Clinical Study Data Request, 5 companies; and Yale University Open Data Access, 1 company), 9 companies (62 trials [20%]) shared through an internal company process, and 21 companies (31 trials [10%]) had no defined process to share IPD.

Eighteen of the top 20 pharmaceutical companies by global revenue sponsored trials in the study sample. These 18 companies owned 81 of the audited medicines for which results from 245 trials (81%) were summarized in their product labels. In addition, for the top 10 anticancer medicines by global revenue, results from 89 trials (29%) were summarized in their respective product labels.

Of the 304 industry-sponsored trials audited, the eligibility for IPD sharing status was publicly available for 64 trials (21%). The remaining 240 trials (79%) required an inquiry to the sponsor to establish whether the trial was eligible for data sharing. The median (IQR) response time to these inquiries was 42 days (7-60 days). For 9 trials (3%) sponsored by 8 different pharmaceutical companies (16% of the sample), no response to the eligibility inquiries was received.

### Eligibility to Share

**Figure 1.**
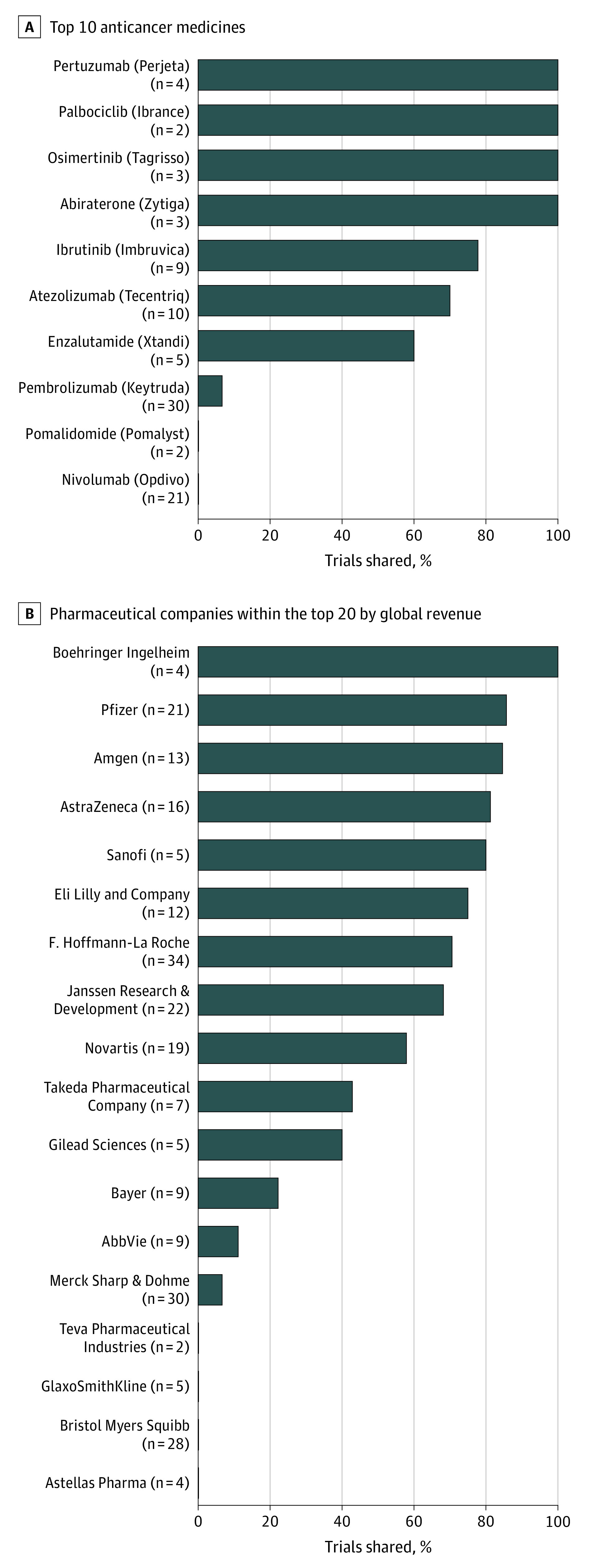
Eligibility to Share Individual Participant Data

Of the 304 included trials, 136 (45%) were eligible for IPD sharing with independent researchers. Figure 1 presents trial IPD sharing eligibility for pharmaceutical companies within the top 20 by global revenue and the top 10 anticancer medicines by global revenue. Of the top 20 pharmaceutical companies by revenue, 4 (AbbVie, Bayer, Gilead Sciences, and Takeda) had less than 50% of their oncology trials available for IPD sharing, and 5 (Astellas Pharma, Bristol Myers Squibb, GlaxoSmithKline, Merck Sharp & Dohme, and Teva Pharmaceutical Industries) had less than 10% available for IPD sharing. Of the top 10 anticancer medicines by global revenue, less than 10% of trials on nivolumab, pembrolizumab, and pomalidomide were available for IPD sharing.

**Figure 2.**
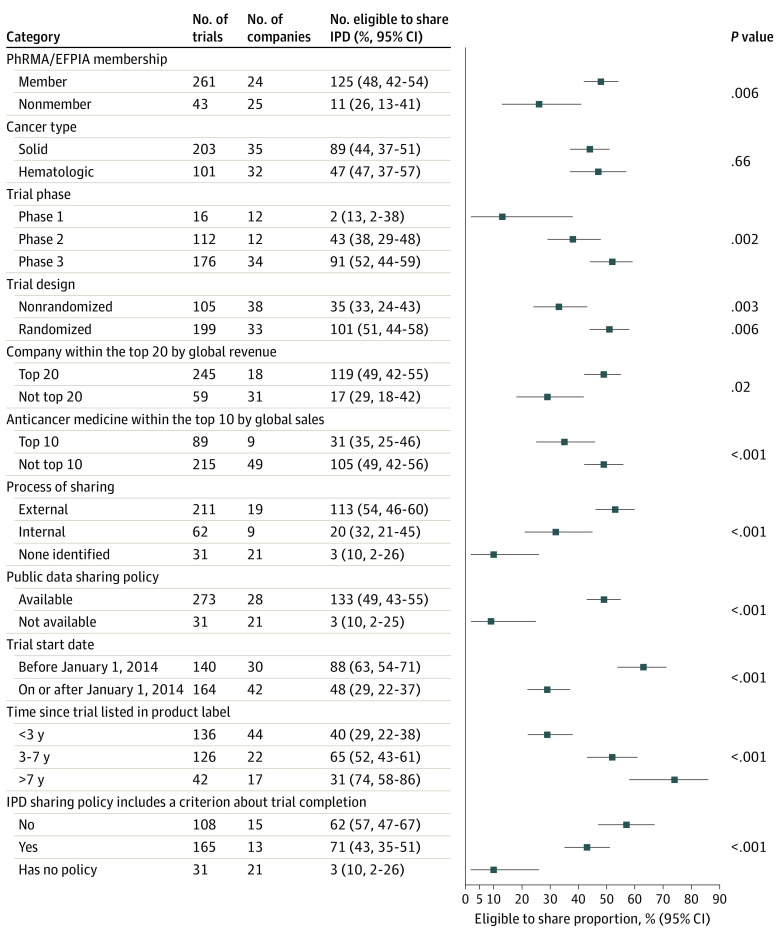
Breakdown of Individual Participant Data (IPD) Sharing Eligibility According to Key Descriptive Subgroups EFPIA indicates European Federation of Pharmaceutical Industries and Associations; PhRMA, Pharmaceutical Research and Manufacturers of America. Whiskers indicate 95% CIs.

Figure 2 and eTable 2 in Supplement 1 present the proportion of trials eligible for IPD sharing according to key descriptive subgroups. Membership in PhRMA/EFPIA (125 [48%] vs 11 [26%]; *P* = .006) and having a publicly available IPD sharing policy (133 [49%] vs 3 [10%]; *P* < .001) were associated with a higher proportion of trials being eligible for IPD sharing. Pharmaceutical companies within the top 20 by global revenue also had a significantly higher proportion of trials eligible for IPD sharing compared with companies outside the top 20 (119 [49%] vs 17 [29%]; *P* = .006), and companies that shared IPD on an external platform had a higher proportion of trials eligible for IPD sharing than companies that shared through an internal process or had no formal process to share IPD (113 [54%] vs 20 [32%] vs 3 [10%], respectively; *P* < .001).

In contrast, the proportion of trials eligible for IPD sharing was significantly lower for the top 10 anticancer medicines by global sales compared with anticancer medicines not in the top 10 (31 [35%] vs 105 [49%]; *P* = .02). The eligibility proportion was lower for nonrandomized trials than for randomized trials (35 [33%] vs 101 [51%]; *P* = .003), phase 1 vs phase 2 and phase 3 trials (2 [13%] vs 43 [38%] vs 91 [52%], respectively; *P* = .002), and trials with a start date after January 1, 2014 (48 [29%] vs 88 [63%] before January 1, 2014; *P* < .001). Eligibility was also lower for trials listed in a product label less than 3 years ago compared with trials listed in a product label 3 to 7 years ago or more than 7 years ago (40 [29%] vs 66 [52%] vs 31 [74%], respectively; *P* < .001). Finally, the proportion of trials eligible for IPD sharing was lower when the sponsoring company had an IPD sharing policy that included a criterion about trial completion compared with companies without such a criterion (71 [43%] vs 62 [57%]; *P* < .001). No difference in IPD sharing eligibility was observed between trials for anticancer medicines used to treat solid tumors and those used for hematologic malignant neoplasms.

Individual participant data were confirmed as ineligible for sharing for 168 (55%) trials (Table). The most common reason communicated for trial IPD sharing ineligibility was that the trial was still ongoing (89 [53%])—that is, the sponsor indicated that follow-up was continuing for the trial and, as such, the IPD for the results reported in the product label were not available for sharing. On ClinicalTrials.gov, the final completion dates for these 89 trials range from 2020 to 2027, with 59 (66%) documented as having passed primary completion and 9 (10%) as fully complete. An additional 21 (13%) were indicated as ineligible for IPD sharing because, despite passing their final completion date, the IPD remained under embargo. The results of 109 anticancer medicines (95%) were summarized in the product labels before passing the follow-up completion date on ClinicalTrials.gov.

**Table.** Breakdown of Reasons for Individual Participant Data (IPD) Sharing Ineligibility as Provided by the Clinical Trial Sponsor

Reason	No. (%)		
	Total cohort (N = 304)	Trials by company within the top 20 by global revenue (n = 245)	Trials for top 10 anticancer medicines by global sales (n = 89)
Trials confirmed as ineligible for IPD sharing	168 (55)	126 (51)	58 (65)
Study still ongoing	89 (53)	75 (60)	37 (64)
Study has passed final completion, but IPD still under embargo	21 (13)	17 (13)	14 (24)
Medicine not approved by both the EMA and FDA or ongoing regulatory submission	12 (7)	9 (7)	2 (3)
Phase 1/2 trials are out of scope	10 (6)	9 (7)	0
Consent form issues	9 (5)	5 (4)	3 (5)
Sponsor does not share IPD	6 (4)	0	0
No response received within 4 mo	9 (5)	0	0
Other^a^	12 (7)	11 (9)	0

Abbreviations: EMA, European Medicines Agency; FDA, US Food and Drug Administration.

^a^ Other reasons were that the study did not fulfill the requirement for data sharing transparency, was of a rare cancer type, was not in standard format, was completed before 2013, required a collaboration agreement, or was of a product approved before 2014.

Because of infrequency in the sample, IPD sharing eligibility of nonindustry-sponsored trials is summarized in eAppendix 2 in Supplement 1. The raw data set generated and analyzed in this study is available in Supplement 2.

## Discussion

To our knowledge, this quality improvement study is the largest structured assessment of IPD sharing eligibility of clinical trials for recently approved medicines and the first to evaluate data sharing for pivotal industry-sponsored oncology trials. In our sample of 304 trials underpinning the FDA approval of 115 new anticancer medicines over the past 10 years, 136 (45%) were confirmed as eligible for IPD sharing and 168 (55%) confirmed as ineligible. With profit correlating to global drug use, the finding that Astellas Pharma, Bristol Myers Squibb, GlaxoSmithKline, Merck Sharp & Dohme, and Teva Pharmaceutical Industries had less than 10% of their sampled oncology trials available for IPD sharing represents a missed opportunity.

Before this study, the largest structured assessments of the eligibility of independent researchers to request industry-sponsored clinical trial IPD were conducted by Murugiah et al^27^ and Hopkins et al.^13^ In 2016, Murugiah et al identified that IPD were eligible for sharing from approximately 25% of large cardiovascular trials, whereas in 2018, Hopkins et al documented that only 15% of clinical trials were available for IPD sharing 2 years after publication of primary results, with no sharing occurring for the oncology trials in the sample. Our study reveals a substantial increase in IPD sharing for major oncology trials, with 45% (136 of 304) of the audited trials confirmed as eligible. Further exemplifying improvements in IPD sharing awareness by the pharmaceutical industry, Hopkins et al did not receive a response to 36% of IPD sharing inquires in 2018, whereas our nonresponse rate was only 3%. In our study, only 32% of evaluated pharmaceutical companies outside the top 20 by global revenue had a publicly accessible data sharing policy, which is in stark contrast to 100% of sponsors within the top 20 by global revenue. These findings are in line with previous research^13,27,28,29^ and demonstrate a continued need for advocacy and support for smaller pharmaceutical companies to enable data transparency. Despite their lower revenue, these smaller companies anchor the registration of a substantial portion of critical innovator medicines in oncology.

In this study, we confirmed that IPD were eligible for sharing from 136 industry-sponsored trials that had results summarized in the product labels of 60 anticancer medicines approved by the FDA over the past 10 years. These trials included more than 70 000 patients and provide an immense opportunity for independent scientific investigations by regulators, clinicians, and researchers. Of note, this opportunity includes IPD sharing for more than 50% of the trials summarized in the product labels of atezolizumab, abiraterone, enzalutamide, ibrutinib, osimertinib, palbociclib, and pertuzumab. Furthermore, 5 of the top 20 pharmaceutical companies by revenue (Amgen, AstraZeneca, Boehringer Ingelheim, Pfizer, and Sanofi) indicated that more than 75% of their sampled oncology trials were eligible for IPD sharing. These noteworthy achievements for transparency were catalyzed by the 2014 PhRMA/EFPIA guiding principles on responsible data sharing^11,12^ and are a beacon of hope to one of the least trusted industries in the world.^4,11^

On the other hand, IPD were unavailable for sharing from 168 investigated industry-sponsored trials that had results summarized in the product labels of 78 anticancer medicines approved by the FDA in the past 10 years. These trials included more than 85 000 participants. It is of great concern that this wealth of anonymized IPD remains unavailable to independent investigation despite the rollout of the medicines within the US and global cancer populations based on the product label results. When participants commit to these trials, they are generally advised and reasonably expect that, although they may not personally benefit from their participation, the knowledge gained will contribute to better care for future patients. Our findings indicate that this commitment to participants in oncology trials is not yet being fully met.

An important strength of this study is that it is the first to have a large enough sample size to facilitate key company-, drug-, and trial-level subgroup evaluations. The trials evaluated were more likely to be eligible for IPD sharing if the medicine owner was a PhRMA/EFPIA member, had a publicly available IPD sharing policy, shared data through an external platform, and were within the top 20 by global revenue. Randomized and phase 3 trials were also more likely to be eligible for IPD sharing. Trials on the top 10 anticancer medicines by global sales, those that were more recently initiated or listed in a product label, and those performed by a sponsor with an IPD sharing policy including a criterion based on trial completion were less likely to be eligible for IPD sharing. Given these findings, it is recommended that in order to improve data sharing, medicine sponsors should (1) join PhRMA/EFPIA and establish a data sharing policy, (2) establish IPD sharing processes external to the company (ie, recognizing that there is an internal conflict of interest), and (3) have a policy that states that all IPD underlying results presented in a product label will be immediately eligible for sharing. The third recommendation aims to remove overly long embargo periods and ensure that registration of a medicine, an event that allows widespread use, immediately triggers sharing of the clinical trial data supporting the registration. However, long-term follow-up of clinical trials remains essential, and sharing of IPD after registration should not jeopardize efforts to collect data on long-term outcomes. Of note, the reason for the unavailability of 89 of the 168 trials ineligible for IPD sharing was that the trial was still ongoing, and it is concerning that, by our estimate, 50% of these trials will still be unavailable for sharing in 2 years. Highlighting the importance of policies that facilitate the sharing of primary outcome data, 109 (95%) of the 115 anticancer medicines evaluated herein at some point had the results of a trial summarized in the product label before passing the follow-up completion date on ClinicalTrials.gov.

A recent editorial in the *British Medical Journal* called for pharmaceutical companies to update their policies to facilitate IPD sharing for newly registered medicines.^4^ The editors emphasized that because of the embargo criterion within the transparency policies of Pfizer and Moderna, IPD underpinning the registration of COVID-19 vaccines will not be available for years, which is not acceptable given the scale and current relevance of their use. We make a similar call to the justifiability of 90% of the trials summarized in the product labels of nivolumab, pembrolizumab, and pomalidomide being ineligible for IPD sharing. These drugs represent major health initiatives that reap substantial profits; therefore, patients deserve the confidence that all opportunities to understand the benefits and harms of the treatment for their conditions have been made and that the scientific claims have been independently scrutinized.

### Limitations

A study limitation is that our sample was focused on anticancer medicines registered by the FDA over the past 10 years. This period was chosen because the product label results are the centerpiece of safety and efficacy for newer medicines, and thus the data should be subject to high scrutiny. However, the generalizability of the findings to older medicines or medicines solely approved by the European Medicines Agency is unknown. Furthermore, because most newer medicines have been developed by an industry sponsor, the sample of nonindustry-sponsored trials was too small for reliable comparison. Inherently, the data sharing practices of nonindustry trial sponsors (eg, academic and health institutions) deserve a purposeful audit. Finally, future studies should investigate the time from proposal submission to data receipt (time to access should be approximately 4 months),^15,16^ data completeness upon receipt, researcher support initiatives (eg, data dictionaries),^10^ and the sharing of data for medicines beyond cancer treatment. Of note, during the 10-year sampling period, the FDA approved 437 novel therapies.

## Conclusions

This quality improvement study revealed that 136 (45%) trials underpinning the FDA approval of 115 anticancer medicines over the past 10 years were indicated as eligible for IPD sharing. This outcome demonstrates a substantial increase in IPD sharing for industry-sponsored oncology trials over the past 5 years and represents a significant resource for scientific discovery. Nonetheless, 55% of the queried trials were confirmed as not available for IPD sharing, and less than 10% of the trials with results summarized in the product labels of nivolumab, pembrolizumab, and pomalidomide were available. Because these trials form the basis of safety and efficacy claims for new medicines, we question whether it is justified that the data are unavailable to independent scrutiny. On the basis of our findings, we reiterate calls that transparency policies need updating so that all IPD that inform results presented in a product label or underpin drug registration are immediately eligible for sharing.

## Notes

Supplement 1. **eAppendix 1.** Standard Request Email **eAppendix 2.** IPD Sharing Eligibility of Nonindustry-Sponsored Trials **eTable 1.** Summary of Trials by Cancer Subtype **eTable 2.** Breakdown of IPD Sharing Eligibility According to Key Descriptive Subgroups

Supplement 2.
Raw Data Set
