# Supplementary material for: Audit of Data Sharing by Pharmaceutical Companies for Anticancer Medicines Approved by the US Food and Drug Administration
Source: JAMA Oncol. 2022 Jul 28;8(9):1310–6. doi: 10.1001/jamaoncol.2022.2867 (PMC9335250; doi:10.1001/jamaoncol.2022.2867)
Supplement: Supplement 1. — eAppendix 1. Standard Request Email eAppendix 2. IPD Sharing Eligibility of Nonindustry-Sponsored Trials eTable 1. Summary of Trials by Cancer Subtype eTable 2. Breakdown of IPD Sharing Eligibility According to Key Descriptive Subgroups [file jamaoncol-e222867-s001.pdf]

Supplemental Online Content

Modi ND, Abuhelwa AY, McKinnon RA, et al. Audit of data sharing by pharmaceutical companies for anticancer medicines approved by the US Food and Drug Administration. *JAMA Oncol*. Published online July 28, 2022. doi:10.1001/jamaoncol.2022.2867

eAppendix 1. Standard Request Email

eAppendix 2. IPD Sharing Eligibility of Nonindustry-Sponsored Trials

eTable 1. Summary of Trials by Cancer Subtype

eTable 2. Breakdown of IPD Sharing Eligibility According to Key Descriptive Subgroups

This supplemental material has been provided by the authors to give readers additional information about their work.

© 2022 Modi ND et al. *JAMA Oncology*.

eAppendix 1. Standard Request Email

Title: Data Availability and Policy   Month, Date, 2021

To whom it may concern,

This is Natansh Modi, a PhD candidate in the Precision Medicine Group at Flinders University, Australia. Our group led by Dr Ash Hopkins is reviewing the availability and policy for sharing of oncology trials for independent scientific research.

I write to request clarification regarding the availability of individual participant data from clinical trials conducted by [COMPANY NAME]. I understand that individual participant data from eligible clinical trials may be shared with qualified researchers to facilitate further scientific research and honour the contribution of trial participants. Specifically, I request confirmation of whether the following clinical trials are eligible (in scope) for sharing of individual participant-level data with a valid research proposal.

1.

2.

If any of these studies are not currently in scope for sharing, I would appreciate details on when the trial(s) will become eligible for sharing individual participant data.

Thank you for your assistance with this query. The responses to this enquiry will be documented and compared to the transparency and policy of other companies. A major facet of honouring the contribution of trial participants is enabling independent research. Only through understanding eligibility for sharing across companies for these specific trials can our plans for future research be validly and timely designed.

Sincerely, Natansh Modi and Dr Ash Hopkins

© 2022 Modi ND et al. *JAMA Oncology.*

eAppendix 2. IPD sharing eligibility of nonindustry-sponsored trials

The results of 14 non-industry-sponsored trials were summarised in the product labels of the audited anticancer medicines which had been approved by the FDA in the 10-year sampling period. These trials were sponsored by 9 different non-industry sponsors. Of the 14 non-industry-sponsored trials, 2 trials were for cytotoxic medicines, 4 for immunomodulators, and 8 for targeted therapeutics not elsewhere specified. Of the 14 non-industry trials audited, 6 (43%) were in patients with solid tumours and 8 (57%) in haematological cancer. There were 7 (50%) phase 2 trials, 6 (43%) phase 3 trials, and for 1 (7%) trial the phase was not documented on clinicaltrials.gov. 10 (71%) trials had a trial start date before 1 Jan 2014, and 4 (29%) trials had a trial start date after 1 Jan 2014.

Of the 14 non-industry-sponsored trials audited, 7 (50%) trials were indicated as eligible for IPD sharing, and 7 (50%) were identified as not available for IPD sharing with independent researchers.

© 2022 Modi ND et al. *JAMA Oncology.*

eTable 1. Summary of Trials by Cancer Subtype

**Cancer subtype**	**Number of trials**
Lung Cancer	39
Leukemia	28
Lymphoma	27
Breast Cancer	26
Myeloma	25
Melanoma	21
Prostate Cancer	15
Bladder Cancer	9
Liver Cancer	8
Colon and Rectal Cancer	8
Kidney Cancer	8
Ovarian Cancer	6
Oesophageal Cancer	4
Stomach Cancer	3
Thyroid Cancer	3
Other Solid Cancers	53
Other Haematological Cancers	21

© 2022 Modi ND et al. *JAMA Oncology*.

eTable 2. Breakdown of IPD Sharing Eligibility According to Key Descriptive Subgroups

Category		Companies (n)	Trial eligible for IPD sharing Yes	No	P-value*
PhRMA/EFPIA membership	Member	24	125 (48%)	136 (52%)	<0.01
	Non-Member	25	11 (26%)	32 (74%)	
Cancer Type	Solid	35	89 (44%)	114 (56%)	0.66
	Haematological	32	47 (47%)	54 (53%)	
Trial Phase	Phase 1	12	2 (13%)	14 (87%)	<0.01
	Phase 2	12	43 (38%)	69 (62%)	
	Phase 3	34	91 (51%)	85 (49%)	
Trial Design	Nonrandomized	38	35 (33%)	70 (67%)	<0.01
	Randomized	33	101 (51%)	98 (49%)	
Company within the top 20 by global revenue	Top 20	18	119 (49%)	126 (51%)	<0.01
	Not Top 20	31	17 (29%)	42 (71%)	
Top 10 anticancer medicine by global sales	Top 10	9	31 (35%)	58 (65%)	0.02
	Not Top 10	49	105 (49%)	110 (51%)	
Process of sharing	External	19	113 (54%)	98 (46%)	<0.01
	Internal	9	20 (32%)	42 (68%)	
	None Identified	21	3 (10%)	28 (90%)	
Public data sharing policy	Available	28	133 (49%)	140 (51%)	<0.01
	Not Available	21	3 (10%)	28 (90%)	
Trial Start Date	< 1 Jan 2014	30	88 (63%)	52 (37%)	<0.01
	≥ 1 Jan 2014	42	48 (29%)	116 (71%)	
Time since trial listed in product label	<3 years	44	40 (29%)	96 (71%)	<0.01
	3-7 years	22	65 (52%)	61 (48%)	
	>7 years	17	31 (74%)	11 (26%)	
IPD sharing policy includes a criterion about trial completion	No	15	62 (57%)	46 (43%)	<0.01
	Yes	13	71 (43%)	94 (57%)	
	Has no policy	21	3 (10%)	28 (90%)	
Chi-sq evaluation of the distribution between trial eligible for IPD sharing "Yes" and "No"					

© 2022 Modi ND et al. *JAMA Oncology.*
